# Effects of (-)-epigallocatechin gallate on RPE cell migration and adhesion

**Published:** 2010-04-03

**Authors:** Chi-Ming Chan, Jheng-Hua Huang, Han-Sun Chiang, Wen-Bin Wu, Hsin-Huang Lin, Jing-Yin Hong, Chi-Feng Hung

**Affiliations:** 1 School of Medicine, Fu Jen Catholic University, Taipei Hsien, Taiwan; 2 Department of Ophthalmology, Cardinal Tien Hospital, Taipei Hsien, Taiwan; 3 Department of Internal Medicine, Cathay General Hospital, Taipei, Taiwan; 4 Department of life science, Fu-Jen Catholic University, Taipei Hsien, Taiwan

## Abstract

**Purpose:**

In diseases such as proliferative vitreoretinopathy (PVR), proliferative diabetic retinopathy (PDR), and age-related macular degeneration (AMD), retinal pigment epithelial (RPE) cells can initiate proliferation and migration and secrete extracellular matrix (ECM) proteins. (-)-Epigallocatechin gallate (EGCG)—a natural anti-oxidant flavonoid that is abundant in green tea—has been shown to suppress the migration and adhesion of many cell types, but its effects on RPE cell migration and adhesion were unknown. Several studies have shown that platelet-derived growth factor (PDGF) enhances proliferation and migration effects on RPE cells in PVR, and that fibronectin is a major ECM component of PVR tissue. Therefore, we investigated the inhibitory effects of EGCG on RPE cell migration induced by PDGF-BB, an isoform of PDGF, and adhesion by fibronectin.

**Methods:**

The migration of RPE cells was detected by an electric cell-substrate impedance sensing (ECIS) migration assay and a Transwell migration assay. Cells were loaded with 2’,7’-bis-(carboxyethyl)-5(6’)-carboxyfluorescein acetoxymethyl ester (BCECF/AM), and their adhesion to fibronectin was examined. The interactions of EGCG with PDGF-BB were analyzed by a dot binding assay. Cytoskeletal reorganization was examined by immunofluorescence microscopy. The PDGF-BB-induced signaling pathways were detected by western blotting.

**Results:**

In the present study, we find that EGCG can inhibit PDGF-BB-induced human RPE cell migration and, in a dose-dependent manner, RPE cell adhesion to fibronectin. Our analysis demonstrates that EGCG does not directly bind to PDGF-BB and the inhibition of EGCG against fibronectin-induced cytoskeletal reorganization is observed. Furthermore, EGCG is shown to suppress PDGF-BB-induced PDGF-β receptors, downstream PI3K/Akt, and MAPK phosphorylation.

**Conclusions:**

Our results provide the first evidence that EGCG is an effective inhibitor of RPE cell migration and adhesion to fibronectin and, therefore, may prevent epiretinal membrane formation.

## Introduction

The retinal pigment epithelium (RPE) plays an essential role in the proper functioning and maintenance of the neural retina. Adult RPE cells are quiescent, differentiated, and reside in the G_o_ phase of the cell cycle. In diseases such as proliferative vitreoretinopathy (PVR) [1], proliferative diabetic retinopathy (PDR) [2], and age-related macular degeneration (AMD) [3], RPE cells can reenter the cell cycle, initiate proliferation and migration, and secrete extracellular matrix proteins. Breakdown of the blood-retinal barrier can expose RPE cells to a variety of growth factors, cytokines, and neurotransmitter compounds in the subretinal space and in the vitreous [4–6], which can trigger the activation of RPE cells. In PVR, RPE cell activation results in epithelial-mesenchymal transition from mitotically inactive epithelial cells to actively dividing fibroblast-like cells with the ability to migrate [7,8]. These alterations result in the formation of contractile epiretinal membranes in the vitreous cavity and on both surfaces of the retina, which is mainly composed of transformed RPE cells and glial cells, and the contraction of these membranes eventually causes retinal detachment and the loss of vision [9]. Proliferative diabetic retinopathy is another proliferative ocular disease correlated with the migration and proliferation of RPE cells [10].

In AMD, newly formed leaky blood vessels from choroidal neovascularization (CNV) eventually penetrate the Bruch membrane and the RPE cell layer, which leads to the accumulation of blood and serum in the subretinal space, causing detachment of the retina and the formation of disciform scars [11,12].Platelet-derived growth factor (PDGF) plays a vital role in angiogenesis and wound healing by promoting the proliferation and migration of mesenchyme derived cells such as fibroblasts, smooth muscle cells, and pericytes [13].

There are four PDGF isoforms (PDGF-A, -B, -C, and -D) that form homodimers or heterodimers (PDGF-AA, -BB, -AB, -CC, and -DD) through disulfide bonds. Ligand binding induces PDGFR-α and -β tyrosine kinase receptor dimerization, resulting in three possible combinations—PDGFR- αα, - αβ, and -ββ—which have different affinities toward the different isoforms of PDGF. Platelet-derived growth factor-A and -B and their receptors are present in RPE and epiretinal membranes from patients with PVR or PDR, and their concentration is elevated in the vitreous of PVR eyes [14–17]. For PDGF-C and -D, through their effects on RPE functions, recent results have revealed their roles in causing PVR [18,19].

Green tea has been shown to have anti-oxidant and anti-inflammatory effects on different types of cells [9]. Extracts of green tea contain (-)-epigallocatechin gallate (EGCG), (-)-epigallocatechin (EGC), (-)-epicatechin gallate (ECG), (-)-epicatechin (EC), and (+)-catechin. Among them, EGCG is the most abundant and the most active component of green tea. It has been shown that EGCG has a protective effect on RPE cells against UVA-induced damage [20] and reduces retinal ischemia/reperfusion injury [21]; EGCG has also been shown to suppress the migration [22–29] and adhesion [30–33] of many cell types, but its effects on RPE cell migration and adhesion are unknown.

Cell migration is a complex biologic process that entails sequential adhesion and release from the substrate, a process in which cell–matrix interactions play a key role [34]. As PDGF-induced RPE cell migration plays a key role in the formation of PVR membranes, we first determine the effects of EGCG on PDGF-BB-induced RPE cell migration using ECIS migration assays and Transwell migration assays. Moreover, because fibronectin is a major ECM component of PVR tissue [35], we investigate whether EGCG prevents RPE cells adhesion to fibronectin. The possible mechanisms involved in RPE cell migration and adhesion are also investigated.

## Methods

### Materials

Protease inhibitors, BSA (BSA), and fibronectin were purchased from Sigma Chemical Co. (St Louis, MO). Antibodies (Ab) raised against phospho-extracellular signal-regulated kinase 1/2 (ERK1/2), phosphor-phosphoinositide 3-kinases (PI3K), PDGF receptor β (PDGFR-β), and β-actin were from Santa Cruz Biotechnology (Santa Cruz, CA). An Ab raised against phospho-PDGFR-β at Tyr^716^ was from Upstate Biotech Inc. (Lake Placid, NY). Antibodies raised against phospho-c-Jun N-terminal kinase (JNK) were from New England Biolabs, Inc. (Beverly, MA). Antibodies for phospho-p38 were from R&D systems, Inc. (Minneapolis, MN). A secondary antibody of antirabbit-HRP was purchased from Santa Cruz Biotechnology. EGCG was purchased from Sigma Chemical Co.

### Cell culture

Adult human retinal pigment epithelial (ARPE19) cells were purchased from Food Industry Research and Development Institute (Hsinchu, Taiwan) and were maintained in DMEM/F12 supplemented with 10% fetal calf serum (GibcoBRL, Invitrogen Life Technologies, Carlsbad, CA), 100 units/ml penicillin, and 100 mg/ml streptomycin (Sigma Chemical Co.). The cells were cultured in a humidified incubator at 37 °C and 5% CO_2_. For most of the experiments, cells reaching a 90%–95% of confluence were starved and synchronized in serum-free DMEM for 24 h before being subjected to further analysis.

### Electric cell-substrate impedance sensing migration assays

Eight-well array culture ware (ECIS 8W1E) consisting of one active electrode (250 μm diameter) and one large area counter electrode (100 μm^2^) per well were purchased from Applied Biophysics (Troy, NY). To study cell behavior with this instrument, cells are grown in culture wells containing gold film surface electrodes, with ordinary culture media serving as the electrolyte. In its normal mode, an approximately constant current source applies an AC signal of 1 μA, usually at 4 kHz, between a small measuring electrode (250 μm diameter) and a large counter electrode. The instrument monitors both the voltage across the electrodes and its phase relative to the applied current. In addition to reporting the total impedance, these data are converted to resistance and capacitance. As the cells attach and spread on the small electrode, their membranes constrict the current and force it to flow beneath and between the cells, resulting in large increases in impedance. The microampere current and the resulting voltage drop of a few millivolts have no measurable effect on the cells and, hence, the monitoring of cell behavior is noninvasive.

The electrode array was placed in an incubator and a medium (200 μl/well) was added to cover the electrodes. The ARPE19 cells were then inoculated at a concentration of 70,000 cells/well in the arrays and incubated for 24 h, while attachment and spreading were followed by means of impedance measurements. The experiments were conducted on wells where the impedance had achieved a steady-state. Then the wells were submitted to an elevated voltage pulse of 40 kHz frequency, 4 V amplitude, and 10 s duration, which led to death and detachment of the cells present on the small active electrode. The medium were then changed to a serum-free cell culture medium with or without PDGF-BB (20 ng/ml) and 10 μM of EGCG. The cells surrounding the small active electrode that have not been subjected to the elevated voltage pulse then migrated inward to replace the killed cells, and the migration was assessed by continuous impedance measurements for 25 h.

### Transwell migration assay

Migration assays with RPE cells were performed using a modified Boyden chamber model (Transwell apparatus; 8.0 μm pore size; Costar,, Cambridge, MA) [36]. The lower face of the polycarbonate filter (Transwell insert) was coated with 0.3 mg of fibronectin for 30 min in the laminar flow hood. The Transwell insert was placed back onto the 24-well plate and the lower chamber was filled with 0.6 ml of DMEM/F12 serum-free medium, with or without 20 ng/ml of PDGF-BB, and 1, 3, and 10 µM of EGCG. The EGCG was dissolved in phosphate buffered saline (PBS). Human RPE cells (5×10^4^ cells/well) in 200 µl medium were plated to the upper chamber. After 5 h of incubation at 37 °C, all non-migrated cells were removed from the upper face of the Transwell membrane with a cotton swab, and migrated cells were fixed and stained with 0.5% toluidine blue in 4% permanganate aldehyde-fuchsin (PAF). Migration was quantified by counting the number of stained cells per 100× field (high power field, HPF) with a phase-contrast microscope (DMIL^®^ ; Leica, Wetzlar, Germany), and 20 HPFs were photographed and counted in each migration.

### Cell adhesion assay

Ninety-six-well plates (Costar) were coated with 50 μl of fibronectin (15 μg/ml) in PBS at 4 °C overnight. After a brief wash with PBS, the plates were blocked with 10% BSA at 37 °C for 1 h. After trypsinization, suspended RPE cells were labeled with BCECF/AM (10 μg/ml) for 30 min at 37 °C. Until it is cleaved by the intracellular esterase to become green fluorescent BCECF, BCECF/AM is intrinsically nonfluorescent. The labeled cells were washed and resuspended in DMEM to a density of 1×10^5^ cells/ml. The suspended cells (95 μl) were incubated with 5 μl of PBS or various concentrations of EGCG for 1 h at 37 °C. After washing twice with PBS and after the addition of a radioimmunoprecipitation assay buffer, the non-adherent cells were removed by aspiration and the 96-well plates were subjected to measurement with a Wallac Victor 3 1420 multilabel counter (Perkin Elmer, Turku, Finland) using excitation and emission wavelengths of 485 and 535 nm, respectively. Eight plates were quantified for each assay.

### Dot binding assay

A nitrocellulose membrane (Bio-Rad Laboratories, Hercules, CA) was soaked in a buffer (25 mM Tris, 192 mM glycine and 20% methanol) for 30 s. Recombinant PDGF-BB (2 μg/ml in 50 μl) was applied to the membrane with a Bio-Dot microfiltration apparatus (Bio-Rad Laboratories, Hercules, CA) by suction. 2.5 μl of PBS, 3 mM and 10 mM of EGCG, and 5 mM of lycopene were directly spotted on the same membrane. The membrane was then blocked with BSA (5% in PBS) for 0.5 h. After washing with PBS, the membrane was incubated with PDGF-BB (0.5 μg/ml) in PBS for 1 h at room temperature (RT). A brief wash was followed, and the membrane was then incubated with anti-PDGF-BB Ab (2 μg/ml in 1% BSA-containing PBS) for 1 h at RT. After another brief wash, the membrane was incubated with horseradish peroxidase-conjugated Ab before being developed by enhanced chemiluminescence (ECL; NEN, Boston, MA).

### Immunofluorescence microscopy

The procedure for staining actin cytoskeleton has previously been described [37]. Briefly, the trypsinized RPE cells were suspended at 37 °C for 30 min, and then pretreated with or without EGCG for an additional 1 h. The cells were then allowed to adhere on glass coverslips that were precoated with fibronectin (15 μg/ml) for 1 h. Afterwards, they were washed, fixed with 1% PAF for 20 min, and permeabilized with 0.1% Triton X-100 for 10 min. After blocking with 3% BSA, the cells were incubated with fluorescein isothiocyanate (FITC)-conjugated phalloidin (1:200; Sigma). The coverslips were mounted under an Olympus BX51 microscope and the immunofluorescence images were taken using a Spot RT Camera System (Diagnostics Instruments, Inc., Sterling Height, MI).

### Cell lysate preparation and western blot analysis

Platelet-derived growth factor-BB (10 ng/ml) was preincubated with a vehicle or EGCG for 30 min and was then added to human RPE cells. The effect of EGCG on PDGFR-β, PI3K, and MAPKs phosphorylation was analyzed by western blotting. Retinal pigment epithelial cells were washed with prechilled PBS and lysed in a radioimmunoprecipitation assay buffer (20 mM Tris–HCl, pH 7.4, 137 mM NaCl, 2 mM EDTA. One mM sodium fluoride, 1% Triton X-100, 0.5% sodium deoxycholate, 0.1% SDS, 10% glycerol, 1 mM sodium orthovanadate, 1 mM PMSF, and 1 μg/ml aprotinin and leupeptin [freshly prepared]). After sonication, the lysate was centrifuged (14,000× g for 15 min at 4 °C) and the supernatant was transferred to a tube. The protein content was quantified with the Pierce protein assay kit (Pierce, Rockford, IL). Total protein was separated by electrophoresis on 10% SDS polyacrylamide gels and the proteins were electroblotted onto polyvinylidene fluoride (PVDF) membranes and probed using the specific antibodies mentioned. Immunoblots were detected by enhanced chemiluminescence (Chemiluminescence Reagent Plus; NEN, Boston, MA). For some of the experiments, the PVDF membrane was stripped at 60 °C for 30 min with a stripping buffer containing 62.5 mM Tris-HCl, pH 6.7, 2% SDS, and 100 mM β-mercaptoethanol.

### Statistical analysis

Data are expressed as mean±standard error (SE), unless otherwise indicated. Comparison of the means of two groups of data are made using an unpaired, two-tailed Student's *t* test. We consider p values <0.05 as statistically significant. The data are analyzed using SigmaPlot for Windows Version 10.0.

## Results

### (-)-Epigallocatechin gallate inhibits platelet-derived growth factor-BB-induced retinal pigment epithelial migration

**Figure 1:**
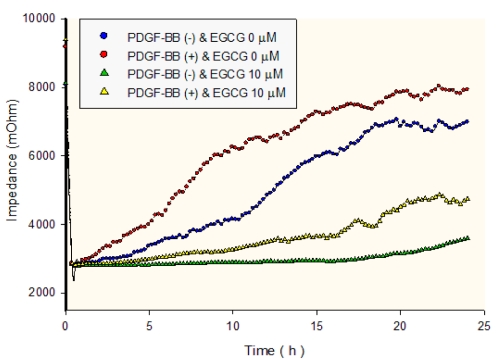
Impedance measurements on retinal pigment epithelium (RPE) cells as a function of time. Cultured adult human retinal pigment epithelial (ARPE19) cells were plated in chambers containing gold electrodes, and impedance measurements were made at fixed time intervals for 24 h. The epigallocatechin gallate (EGCG) inhibited ARPE19 cell migration, even without the presence of platelet-derived growth factor (PDGF)-BB. For the cells treated with 20 ng/ml PDGF-BB, the impedance increased faster and was significantly higher at 24 h than the cells treated without 20 ng/ml PDGF-BB (p<0.05) or with 10 μM EGCG (p<0.05). The experiment was performed four times with similar results. Each value represents the mean of four replicates.

In the ECIS migration assays, cultured RPE cells were plated in chambers containing gold electrodes, and time-dependent impedance measurements were made (Figure 1). When incubated with PDGF-BB, ARPE19 cell migration was stimulated and the impedance (7933±305 mOhm at 24 h) increased faster and was significantly larger compared to cells without PDGF-BB (6989±491 mOhm at 24 h, p<0.05). (-)-Epigallocatechin gallate inhibited ARPE19 cell migration even in the absence of PDGF-BB (6989±491mOhm without EGCG and without PDGF-BB versus 3600±238mOhm with EGCG at 24 h and without PDGF-BB, n=4, p<0.05). For ARPE 19 cells treated with PDGF-BB, the impedance (7933±305 mOhm at 24 h) was significantly reduced with the addition of 10 μM EGCG (4743±370 mOhm at 24 h, p<0.05), indicating that EGCG inhibits PDGF-BB-induced RPE cell migration.

**Figure 2:**
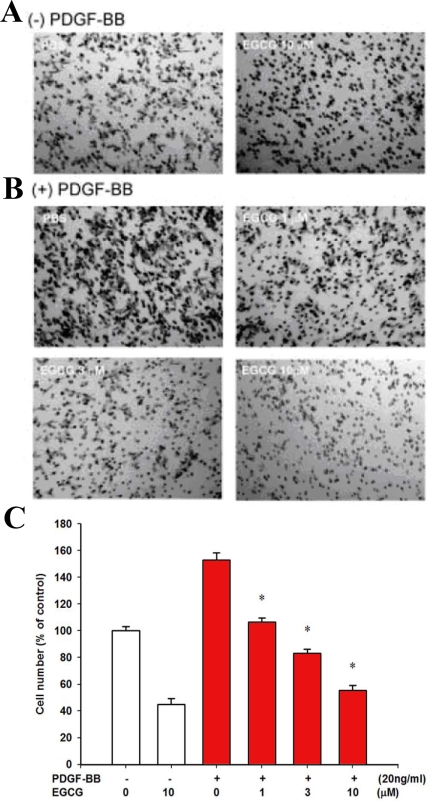
Effects of epigallocatechin gallate (EGCG) on retinal pigment epithelium (RPE) cell migration. **A**, **B**: Epigallocatechin gallate inhibits platelet-derived growth factor (PDGF)-BB-induced RPE cell migration. Transwell inserts were coated with fibronectin. Human RPE cells were seeded in the upper chamber in the presence of vehicle or EGCG. The inserts were assembled in the lower chamber, which was filled with serum-free ([−]PDGF-BB; **A**) or PDGF-BB-containing medium ([+]PDGF-BB; **B**) and preincubated with a vehicle or EGCG on the polycarbonate filter of the insert for 30 min. PBS, without PDGF-BB or EGCG, served as the control (**A**, left). Human RPE cells that migrated to the underside of filter membrane were photographed and counted in high-power field (HPF, magnification, 100×) under a phase-contrast light microscope. The scale bar represents 100 μm. The black spots are the pores of the Transwell membrane and the grayish fusiform cells are the ARPE cells. **C**: Quantitative analysis of migrated cells. Twenty HPFs were counted in each migration. All experiments were conducted in duplicate and similar results were obtained at least two to three times. Data are presented as percent of control (the first unfilled bar, PBS only) in cell counts. *p<0.05 significantly differs from PDGF-BB-stimulated cells (the first filled bar).

In the Transwell migration assay, coating with the fibronectin alone was observed to induce only a small amount of ARPE19 cell migration when compared to samples without the coating (data not shown).Without EGCG or PDGF-BB, PBS served as the control. Significant RPE cell migration was observed after PDGF-BB stimulation for 5 h at 37 °C (153±5.6% of control; Figure 2). We found that EGCG suppressed RPE cell migration without PDGF-BB (Figure 2A). Quantitative analysis indicates that in the absence of PDGF-BB, 55% of migration (45±4.2% of control) was inhibited by 10 μM of EGCG (Figure 2C). Moreover, EGCG suppressed PDGF-BB-induced RPE cell migration in a dose-dependent manner (106.4±3.1%, 83.1±3.2% and 55.3±3.7% of control for 1, 3 and 10 μM of EGCG, respectively; Figure 2B). A total of 64% of PDGF-BB-induced RPE cell migration (153±5.6% versus 55.3±3.7% of control, p<0.05) was inhibited by 10 μM of EGCG (Figure 2C). Therefore, the Transwell migration assay demonstrates that EGCG inhibits PDGF-BB-induced RPE cell migration (n=8).

### (-)-Epigallocatechin gallate inhibits retinal pigment epithelial cell adhesion to fibronectin

**Figure 3:**
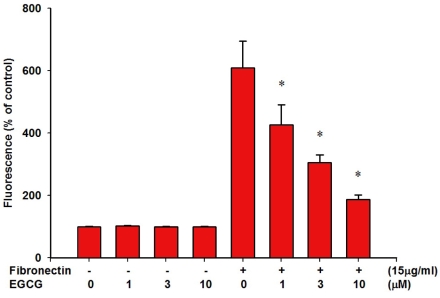
Epigallocatechin gallate (EGCG) inhibited human retinal pigment epithelium (RPE) cell adhesion to fibronectin in a dose-dependent manner. Suspended RPE cells were loaded with 2’,7’-bis-(carboxyethyl)-5(6’)-carboxyfluorescein acetoxymethyl ester (BCECF/AM) and pretreated with different concentrations of EGCG. The cells were added to 96-well plates precoated with fibronectin (15 μg/ml) and incubated for 1 h at 37 °C. Cell adhesion was then measured by a fluorescence plate reader. Eight plates were quantified for each assay. Results are expressed as fluorescence intensity and represented by mean±SEM. *p<0.05 significantly differs from platelet-derived growth factor (PDGF)-BB-stimulated cells (the fifth bar).

We further studied the inhibitory effects of EGCG on RPE cell adhesion to fibronectin. Figure 3 shows that RPE cell adhesion did not take place without fibronectin and was only activated when fibronectin was added (609.2±86.1% of control). The addition of ECGC significantly inhibited RPE cell adhesion in a dose- dependent manner (426.5±64.1%, 304.8±25.7%, and 187.5±13.6% of control at 1, 3, and 10 μM, respectively, p<0.05, n=5).

### (-)-Epigallocatechin gallate does not directly bind to platelet-derived growth factor-BB in dot binding assay

**Figure 4:**
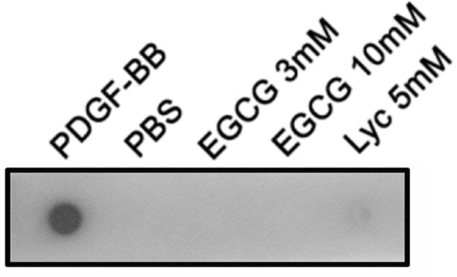
Epigallocatechin gallate (EGCG) cannot directly interact with platelet-derived growth factor (PDGF)–BB in dot binding assay. Human recombinant PDGF-BB, phosphate buffer saline (PBS), the indicated concentrations of EGCG, and lycopene (Lyc) were applied onto the nitrocellulose (NC) membrane. The membrane was incubated with PDGF-BB in PBS and then developed by probing with Ab directed against PDGF-BB. Epigallocatechin gallate cannot directly interact with PDGF-BB, but lycopene can. The results presented are representative of three independent experiments.

Recombinant human PDGF-BB, EGCG, and lycopene were immobilized on the nitrocellulose (NC) membrane. After incubation with or without PDGF-BB, the membrane was further incubated with antibodies against PDGF-BB and then developed. We observed that immobilized PDGF-BB can be recognized by the anti-PDGF-BB Ab, suggesting the specificity of Ab. The 3 mM and 10 mM of EGCG did not directly bind to PDGF-BB, but a positive binding signal was detected on the lycopene spot incubated with PDGF-BB. The data indicates that EGCG cannot directly bind to PDGF-BB (Figure 4).

### (-)-Epigallocatechin gallate changes actin cytoskeleton organization during cell adhesion

**Figure 5:**
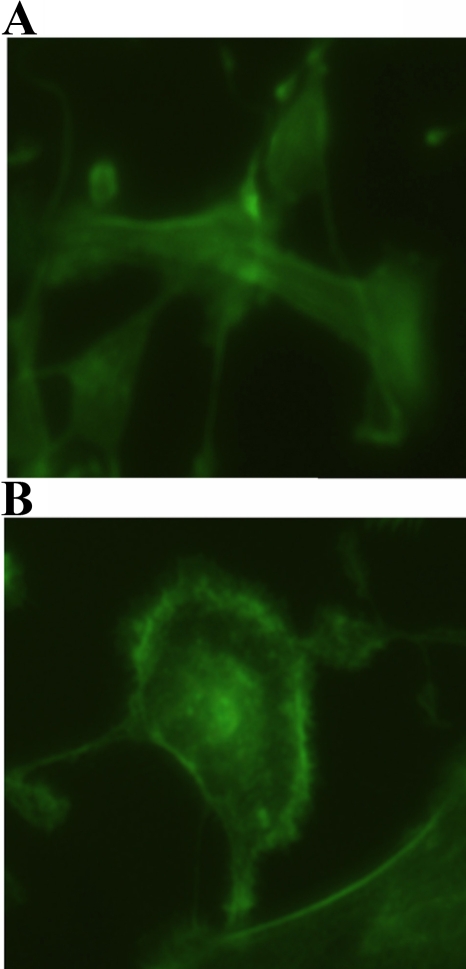
Inhibition of fibronectin-induced human retinal pigment epithelial (RPE) cell cytoskeletal reorganization by Epigallocatechin gallate (EGCG). Suspended RPE cells pretreated with phosphate buffer saline (PBS, control) or 10 μM EGCG for 1 h were seeded and allowed to adhere on collagen-precoated glass coverslips for an additional 1 h. After fixation, permeabilization, and blocking with 3% BSA (BSA), cells on coverslips were incubated with fluorescein isothiocyanate (FITC)-phalloidin. Mounted cells were analyzed and photographed under a microscope. Actin formed in adherent cells (**A**), but there was a modification of cytoskeletal reorganization in EGCG-treated cells (**B**).

We next determined if actin cytoskeleton organization during cell adhesion would be affected by EGCG. Our immunofluorescence microscopy confirmed that fibronectin-adherent RPE cells spread well. Typical long stress fibers running across the cell body were observed (Figure 5A). In the presence of EGCG, the changes in the organization of actin cytoskeleton, indicated by the disappearance of the stress fibers and the generation of protrusions at the cell periphery, were prominent (Figure 5B).

### (-)-Epigallocatechin gallate inhibits platelet-derived growth factor-BB-induced platelet-derived growth factor receptor-β, downstream PI3K/Akt and MAPK phosphorylation

**Figure 6:**
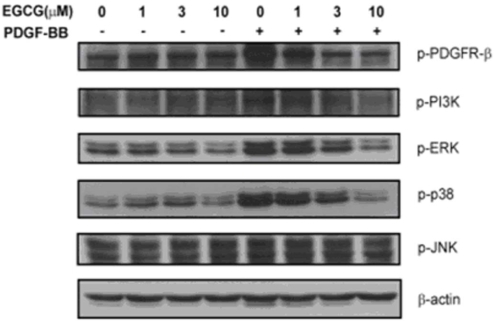
Effects of epigallocatechin gallate (EGCG) on PDGFR-β, PI3K, and MAPKs phosphorylation in human retinal pigment epithelium (RPE) cells. Platelet-derived growth factor-BB (10 ng/ml) was preincubated with a vehicle or 1, 3, and 10 μM of EGCG for 30 min and then added to human RPE cells. Western blotting was used to analyze PDGFR-β, PI3K, and MAPKs phosphorylation. All concentrations of EGCG at or above 3 μM significantly inhibited PDGF-BB-induced PI3K/Akt, ERK1/2, and p38 phosphorylation in human RPE cells in a concentration-dependent manner. However, there was no effect of PDGF-BB and EGCG on c-Jun N-terminal kinases (JNK) phosphorylation. The results presented are representative of four independent experiments.

It has been reported that PDGF-BB binding to a PDGF receptor (PDGFR) is associated with dimerization, autophosphorylation, and activation of PDGFR-tyrosine kinase activity [38], which subsequently causes RPE cell migration through activation of PI3K/Akt and MAPKs signaling [39]. To determine whether a PDGF-BB-induced signaling pathway is affected by EGCG in human RPE cells, the extent of phosphorylation of PDGFR-β and its downstream components was examined. We observed that stimulation of RPE cells with PDGF-BB results in PDGFR-β phosphorylation, as determined by western blotting with Abs directed against phosphotyrosine and PDGFR-β at Tyr^716^ (Figure 6). All concentrations of EGCG at or above 3 μM significantly inhibited PDGF-BB-induced PI3K/Akt, ERK1/2, and p38 phosphorylation in human RPE cells in a concentration-dependent manner. However, JNK phosphorylation was not significantly affected by EGCG.

## Discussion

The human retinal pigment epithelium is composed of highly specialized epithelial cells that are important for retinal homeostasis. The cells adapt to the increase in retinal areas associated with growth and age, but have limited proliferative capacity. However, under pathological conditions, such as PVR, these cells acquire the ability to migrate and proliferate. In PVR, the RPE cells migrate through the retina and come in contact with the vitreoretinal interface, where they proliferate and form traction epiretinal membranes [40–42]. Moreover, in patients with PVR, the PDGF level is elevated in the vitreous and is expressed by RPE and glial cells within PVR membranes [5,43–50]. The contraction of the epiretinal membrane or vitreous constitutes a major contribution to retinal detachment in the later stages of PVR, while PDGF enhances the contraction of fibroblasts [51] and RPE cells [46,52,53]. The increased expression of PDGF-B in the retina enhances the formation of epiretinal membranes, and traction retinal detachment is an important feature of proliferative retinopathy [53]. Furthermore, PDGF-BB shows strong stimulatory effects on the proliferation and migration of RPE cells [19].

In this study, we investigated the effects of EGCG on PDGF-BB-induced cell migration and adhesion of human RPE cells. The inhibitory effect of EGCG on PDGF-BB induced migration is shown by the ECIS migration assays and the Transwell migration assays. Moreover, EGCG can inhibit RPE cell adhesion to fibronectin in a dose-dependent manner. Our previous studies have shown that lycopene, a kind of carotene with inhibitory effects on the RPE cell migration [54], binds to PDGF-BB in dot binding assay [36,55]. However, this study demonstrates that EGCG does not directly bind to PDGF-BB. Cytoskeletal reorganization is considered critical for the adhesion of RPE cells, and our results show that EGCG changes actin cytoskeleton organization during cell adhesion. Furthermore, we found that EGCG suppresses PDGF-BB-induced PDGFR-β, downstream PI3K/Akt, and MARK phosphorylation.

Our results from the dot binding assay demonstrate that EGCG does not directly bind to PDGF-BB while lycopene does. Therefore, we can exclude the effect of the direct binding between PDGF-BB and EGCG. EGCG, however, may bind to the PDGF receptors and affect the cytoskeletal reorganization and the PDGF downstream signaling in RPE cells. The exact mechanism behind this effect of EGCG is now being investigated in our laboratory

As the regulation of cell cytoskeleton organization plays a key role during cell adhesion, we examined the effects of EGCG on fibronectin-induced RPE cell adhesion. In our study, the inhibitory effects of EGCG against fibronectin-induced cytoskeletal reorganization during RPE cell adhesion were observed using immunofluorescence imaging. This finding is consistent with the inhibitory effects of EGCG on RPE cell adhesion to fibronectin.

Previous studies have shown the relatively high expression of PDGFR-β compared to PDGFR-α and the absence of a stimulatory effect of PDGF-AA suggest an important role of PDGFR-β in the activation of the signaling pathways leading to RPE cell migration and proliferation [19]. Since the PI3K-Akt pathway is involved in all cell responses investigated (production of vascular endothelial growth factor [VEGF], cell migration, and proliferation), inhibition of this pathway may be a promising method to block RPE cell responses in proliferative retinopathies. This conclusion is in accordance with previous studies, suggesting a central role of PI3K in experimental PVR [56]. In RPE cells, PDGF is known to evoke activation of PI3K [39,56,57], ERK1/2 [39,58], and P38 [39] signaling. Our results show that PDGF activates three signal transduction pathways in RPE cells, namely, the MEK-ERK1/2, p38 MAPK, and PI3K-Akt pathways. These pathways differentially regulate PDGF-evoked cell responses. In our study, all concentrations above 3 μM EGCG significantly inhibited PDGF-BB-induced PI3K/Akt, ERK1/2, and p38 phosphorylation in human RPE cells in a concentration-dependent manner. However, JNK phosphorylation was not significantly affected by EGCG.

Therapeutic management of PVR is an important issue. Currently, the only effective therapy is to surgically remove the membranes. Furthermore, most clinicians use systemic corticosteroid to inhibit cell proliferation even though the effect is not always beneficial. Recently, there have been several published studies that demonstrate that certain substances have an inhibiting effect on the proliferation of RPE cells in vitro [59–63]. However, the possible toxic side effects restrict the usefulness of these substances as treatment for PVR. EGCG, on the other hand, is a major polyphenolic compound from green tea, which is one of the most widely consumed beverages in the world and has been shown to promote anti-tumor activities such as inhibiting adhesion, migration, and proliferation of tumor cells [37]. This study provides the first evidence that EGCG also inhibits human RPE cell migration and adhesion. Additional studies are required to determine whether EGCG may prevent PVR or epiretinal membrane formation.
